# Novel application of robot-guided stereotactic technique on biopsy diagnosis of intracranial lesions

**DOI:** 10.3389/fneur.2023.1173776

**Published:** 2023-07-27

**Authors:** Yan Feng, Wang Yaming, Shan Yongzhi, Wei Penghu, Wang Hong, Fan Xiaotong, Wang Changming, Chen Sichang, Zhao Guoguang

**Affiliations:** ^1^Department of Neurosurgery, Xuanwu Hospital Capital Medical University, Beijing, China; ^2^China International Neuroscience Institute (China-INI), Beijing, China; ^3^China National Medical Center for Neurological Diseases, Beijing, China; ^4^Precision Diagnosis and Treatment Center for Nervous System Diseases, Xuanwu Hospital Capital Medical University, Beijing, China; ^5^Institute of Biomedical Engineering, Chinese Academy of Medical Sciences & Peking Union Medical College, Tianjin, China

**Keywords:** robotics, brain biopsy, stereotactic surgery, diagnostic rate, influencing factors

## Abstract

**Introduction:**

This study was performed to examine whether there is a link between the application of three types of robot-guided stereotactic biopsy techniques and the diagnostic rate of intracranial lesion biopsy.

**Methods:**

The study involved 407 patients who underwent robot-guided stereotactic intracranial lesion biopsy at Xuanwu Hospital of Capital Medical University from January 2019 to December 2021. Age, sex, lesion characteristics, lesion distribution, surgical method, and target path depth were assessed for their impact on the biopsy diagnostic rate.

**Results:**

The patients’ mean age was 42.1 years (range, 6 months–82 years). All patients underwent robot-assisted stereotactic brain biopsy using one of three different systems: a ROSA robotic system (*n*=35), the CAS-R-2 (*n*=65), or the REMEBOT domestic robotic system (*n*=307). No significant difference was found in the diagnostic rate of positive histopathological findings or the mean time of surgery among the three biopsy modalities. The diagnostic rate was 93.86%. Multiple linear regression analysis showed that age, sex, and biopsy modality did not affect the diagnostic rate *n*>0.05), whereas enhancing lesions and smaller-volume lesions (≤l cm3) were significantly correlated with the diagnostic rate (*p* = 0.01). Lesions located in the suprasellar and pineal regions were significantly associated with the negative diagnostic rate (*p*<0.05).

**Conclusion:**

The presence of enhancing lesions, lesion location, and lesion volume significantly affected the diagnostic rate of brain biopsy. Age, sex, lesion depth, and biopsy modality did not significantly affect the diagnostic rate. All three procedures had high safety and effectiveness.

## Introduction

**Figure 1:**
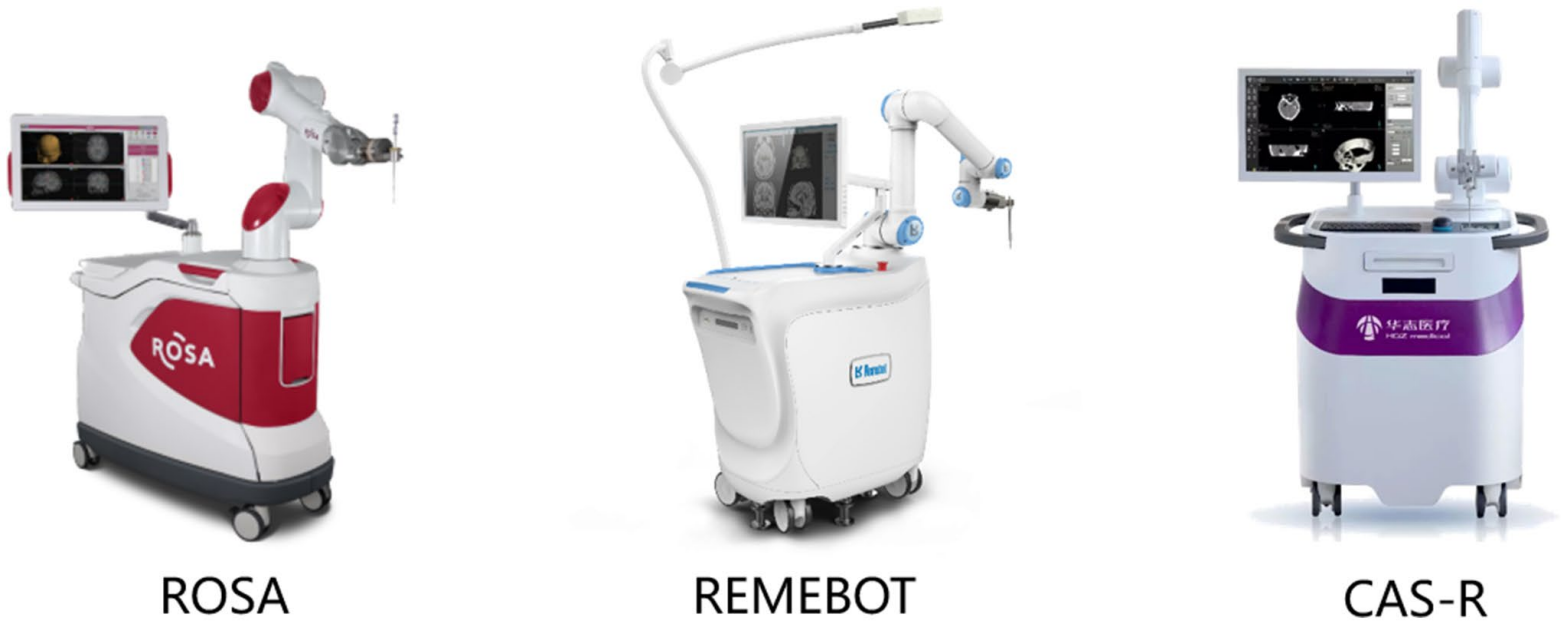
All patients underwent robot-assisted stereotactic brain biopsy using one of three systems: a ROSA robotic system (Zimmer Biomet Robotics, Montpellier, France), the CAS-R-2 (Tianjin Huazhi Technology Co., Ltd., Tianjin, China), or the REMEBOT domestic neurosurgical robot (Beijing Baihui Weikang Technology Co., Ltd., Beijing, China). The ROSA system is housed on a mobile wheeled platform that is rigidly coupled to the skull clamp during surgery. The ROSA can be used in frame-based or frameless modes and incorporates intraoperative imaging into its workflow. The ROSA can use facial laser scanning for registration; i.e., it automatically performs a face scan followed by image reformation. The REMEBOT robot system consists of one arm with six degrees of freedom, one master computer, and one binocular camera. The CAS-R-2 system is mainly composed of five parts: a computer-aided surgical planning system, positioning navigation system, manipulator with five degrees of freedom, platform locking control installation system, and marker recognition and fixation system.

Stereotactic biopsy is an effective surgical procedure that provides a histological diagnosis of intracranial lesions with the advantages of precision, high efficiency, and minimal invasiveness. Stereotactic biopsy plays a vital role in distinguishing among brain tumors, radiation necrosis, inflammation, and other lesions. For instance, histological diagnosis of glioma or lymphoma is vital for decisions regarding subsequent therapy, including the operation strategy, chemotherapy, radiotherapy, and targeted drug therapy. In particular, stereotactic biopsy can be used for multiple lesions; for difficult craniotomy; in high-risk locations such as the suprasellar, pineal, and brainstem regions; and in patients who cannot tolerate surgery because of poor health. With the rapid progress in neuroimaging, neurosurgical navigation technology, and especially the application of neurosurgical robots, stereotactic biopsy techniques for intracranial lesions have been rapidly developed with high visualization, automation, and precision (1–3). Although some researchers have concluded that stereotactic brain biopsy is feasible and safe, few have focused on diverse robotic systems applied *in vivo* (1, 4). In the present study, we established a series of unique treatment groups. We first evaluated a large series of patients who underwent three different types of robot-assisted stereotactic biopsy. We then analyzed our 3-year experience with robot-assisted stereotactic biopsies of brain lesions and confirmed the diagnostic value, predictive factors, and safety of different surgical procedures. As a participating institution in the national neurosurgical robot application project, our center uses a variety of domestic and foreign neurosurgical robot-guided stereotactic surgery systems in the practice of intracranial lesion biopsy (Figure 1). This study was performed to evaluate and analyze the possible factors influencing the diagnostic rate and complications of robot-guided stereotactic brain biopsy in our center. We investigated 407 patients with complete clinical data to identify predictive factors and safety, and we performed a retrospective efficacy analysis of the positive diagnostic rate of robot-assisted stereotactic intracranial lesion biopsy.

## Patients and methods

### Clinical data

This retrospective study involved 407 patients who underwent robot-guided stereotactic intracranial lesion biopsy at Xuanwu Hospital of Capital Medical University, China from January 2019 to December 2021. They comprised 233 male and 174 female patients with a mean age of 42.1 years (range, 6 months to 82 years). Biopsy was performed with a ROSA robotic system (Zimmer Biomet Robotics, Montpellier, France) in 35 patients, the CAS-R-2 (Tianjin Huazhi Technology Co., Ltd., Tianjin, China) in 65 patients, and the REMEBOT domestic neurosurgical robot (Beijing Baihui Weikang Technology Co., Ltd., Beijing, China) in 307 patients. The hospital ethics committee approved the research, and all patients or their relatives provided written informed consent.

### Preoperative preparation

The decision to perform a brain biopsy and the planning of the surgical technique were based on a joint multidisciplinary consultation organized by the Xuanwu Hospital Centre for the Precision Treatment of Difficult Neurological Disorders. The biopsy plan was completed before the surgery. All patients underwent a preoperative magnetic resonance-enhanced thin scan (Contrast-enhanced multiplanar reformation). The volume and target depth were measured by a software outlining method when the lesion was taken.

### Surgical approach of robot-guided stereotactic brain lesion biopsy

Three frameless robotic stereotactic systems are used in our center. The ROSA and REMEBOT are active arm robot systems with six degrees of freedom of movement. The CAS-R-2 is a passive mechanical arm robot system with five degrees of freedom. Robot-assisted stereotactic biopsy systems mainly consist of four key components: an operation planning subsystem, surgical localization subsystem, optical position tracking sensor, and operation subsystem. The operation planning system provides surgeons with a simple, true-to-life, high-performance software tool that generally fulfills the following functions: establishing and maintaining a case history, entering and demonstrating data in Digital Imaging and Communications in Medicine format, performing three-dimensional reconstruction for visualization, and planning the puncture tract. A camera executes the functions of visual registration and visual navigation, and controller and executing instruments perform the function of precisely positioning the puncture needle. The mechanical arm substitutes for a stereotactic arc and is used to hold the probe with which the surgeon finally performs the stereotactic procedure. The surgeon performs depth measurements using the depth of the probe.

**Figure 2:**
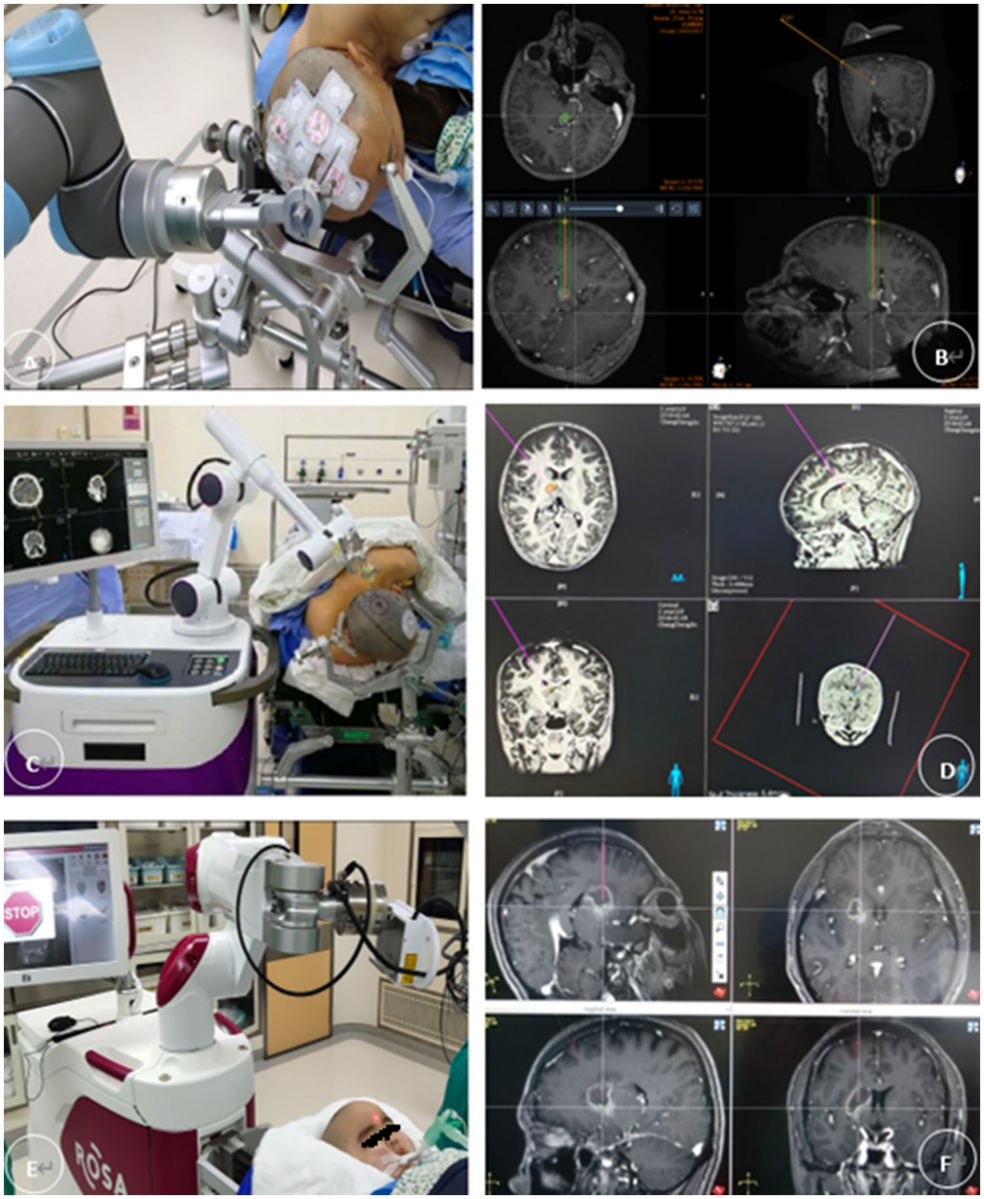
**(A,B)** The REMEBOT and **(C,D)** the CAS-R-2 use scalp markers for registration. The patient’s head is prepared for skin adhesion to the scalp markers before surgery **(E,F)**. The ROSA robot uses facial laser scanning for registration; it automatically performs a face scan followed by image reformation. Magnetic resonance imaging and computed tomography of the head are performed and fused with the surgical planning procedure, and the images are transmitted to the surgical robotic procedure planning system. This system is capable of establishing and maintaining a case history, entering and demonstrating data in Digital Imaging and Communications in Medicine format, performing three-dimensional reconstruction for visualization, and planning the puncture tract. The patient’s head is fixed with a plastic pillow or Mayfield head frame after induction of general anesthesia. The mechanical arm substitutes for a stereotactic arc and is used to hold the probe with which the surgeon finally performs the stereotactic procedure. The surgeon performs depth measurements using the depth of the probe.

The ROSA robot uses facial laser scanning for registration. Specifically, after successful induction of general anesthesia, the head frame is fixed and secured to the robot trolley, manual registration is performed, and the laser device is used to correspond the preoperative computed tomography (CT) reconstruction of the face to the intraoperative bony anatomical landmarks of the face such as the nasal root, inner and outer canthi, brow arch, and other structures. After successful registration, the robot automatically performs a face scan including approximately 5,000–8,000 scan points on the face, and image reformation is then performed. Brain magnetic resonance imaging is performed the day before the CAS-R-2 and REMEBOT procedure, and the images are transmitted to the surgical robotic procedure planning system. The patient must be prepared for skin adhesion to the scalp marker. The markers covered the target lesions and were arranged in a spatially staggered manner so that the three points were not in one plane. Head CT is performed and fused with the surgical plan from the previous surgery. The biopsy target, cranial entry point and biopsy trajectory were designed according to the lesion sites, sizes and shapes determined from three-dimensional imaging. The surgery approach was appropriately designed after three-dimensional reconstruction of the lesions by the their respective stereotactic surgery planning system (5–7). The patient’s head is fixed with a plastic pillow or Mayfield head frame for manual alignment after induction of general anesthesia (Figure 2). Various robotic re-registrations are made, and the alignment is completed with an accuracy error within 2 mm before the brain biopsy procedure is performed. After completing the registration stage, we will disinfect according to the standard surgery procedure and lay out a sterile draping. The operating end of the robotic arm is the fixator through which the cranial drill for puncture and the puncture needle are passed. The cranial drill is a 3-mm-diameter drill. A Sedan aspiration biopsy needle with a lateral cutout is used to obtain the biopsy specimen by negative-pressure aspiration using a 5-mL syringe. Three biopsies are routinely obtained from the target lesion through a single tract or multiple tracts. One-third of the specimens are first sent for intraoperative freezing. The biopsy core is then removed and the biopsy tract is observed for active bleeding while awaiting the return of intraoperative freezing results. The remaining specimens are routinely sent for histopathological examination after the procedure.

### Safety and efficacy evaluation

**Figure 3:**
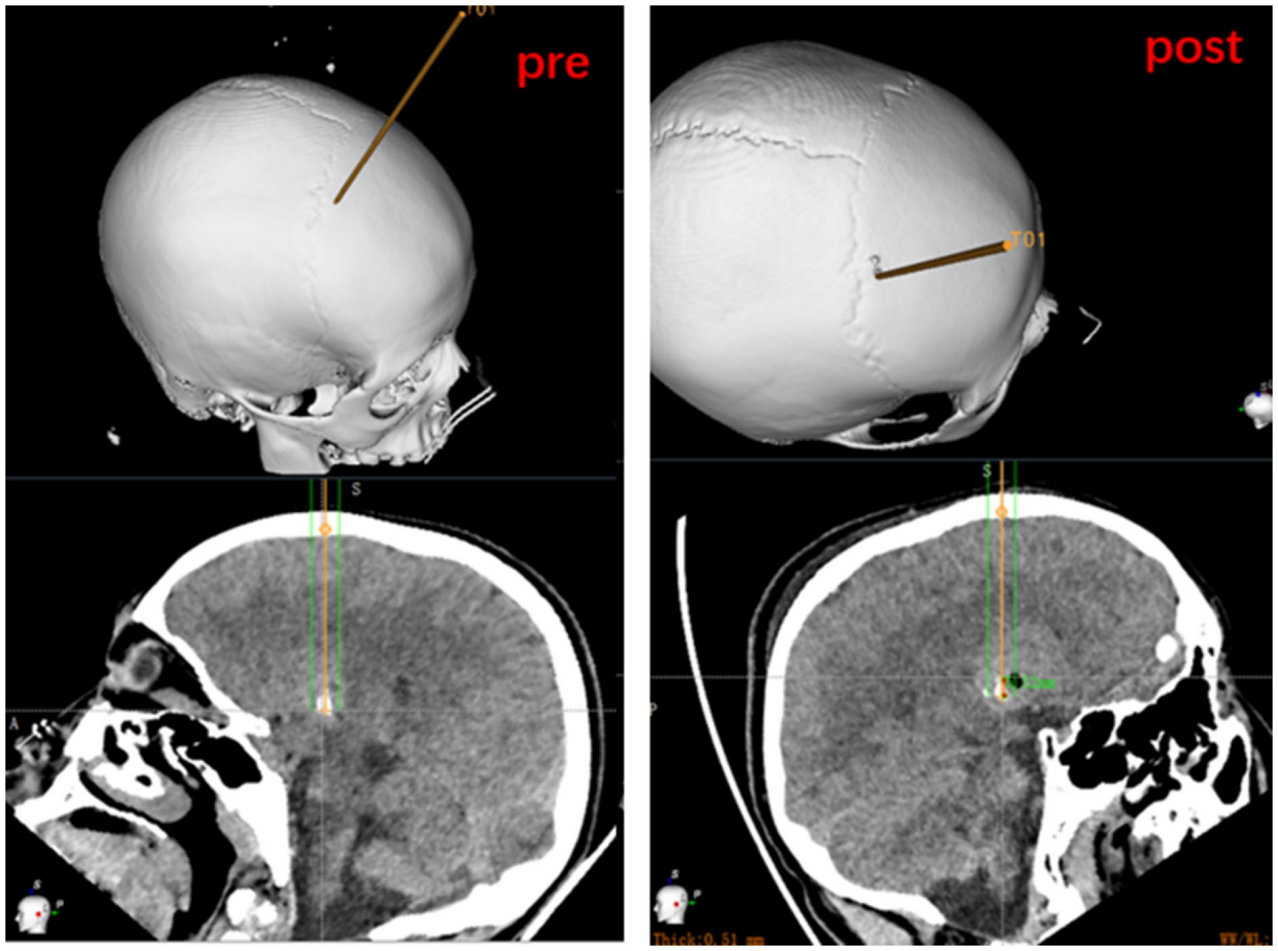
Measurement of entry point and target point error based on preoperatively planned target and on the fusion of postoperative CT to the preoperative dataset.

The cranial CT images are routinely reviewed on postoperative day 1 to determine the status of the path within the target lesion target. A puncture tract or air bubble shadow is usually visible early after the biopsy, helping to determine the accuracy of the extraction site and rule out surgical complications such as intracranial hemorrhage. The postoperative CT images are fused with the preoperatively planned target, and measurements of entry point and target point are compared to calculate error values. The yellow lines represent the biopsy trajectory planned preoperatively. The cross at the end of yellow lines represent the target point. Burr hole is the actual biopsy trajectory of the operation. Air bubble shadow is the real target. Because that three-dimensional (3D) visualization technology of cranial bone and take the target path as the axis with rotate 360 degrees of each level as the average of the measurement of the entry point and target point error (Figure 3).

### Statistical analysis

The factor variables, diagnostic rates, and validity of robot-guided brain biopsies were retrospectively analyzed. Demographic information; clinical, imaging, and histopathological findings; and information on the different biopsy modalities, duration of the procedure, and postoperative complications were collected for all patients. The t-test was performed, and the odds ratio and 95% confidence interval were calculated. Multiple linear regression was used to determine the correlates affecting the rate of diagnosis. A *p* value of <0.05 was considered statistically significant.

## Results

### Procedural outcomes and complications

All patients in this study had a CT scan within the first 24–48 h after the biopsy, which was matched with preoperative images to verify the accuracy of puncture, with a positive diagnostic rate of 93.86% (25/407). Twenty-five patients had negative diagnostic findings (inconclusive in 22 and normal brain tissue in 3) (Table 1). Five patients underwent a second stereotactic biopsy. Four underwent an open biopsy, and a successful histological diagnosis was established. The remaining 10 patients failed to undergo re-biopsy, 5 of these patients were treated by default, and 2 were treated empirically with steroids. The overall postoperative complication rate was 0.98%, and the most notable complications were bleeding at the biopsy site in three patients and cerebral edema in one. Two patients had asymptomatic bleeding (<10 mL) at the puncture site on postoperative CT. No increase in bleeding was seen on re-examination, and no special treatment was given.

**Table 1:** Distribution of brain biopsy lesions, surgical complications and histopathological findings in 407 cases.

Lesion characteristics	Number of cases
Distribution	
**Midline or deep region**	**208**
Brainstem, pontine arm region	49
Basal ganglia area	48
Thalamic region	38
Corpus callosum area	16
Pineal region	11
Paraventricular area	36
Sellar region	10
**Superfical region**	**199**
Lobe of the brain	170
Cerebellum	29
**Complications**	**4**
Bleeding	3
Edema	1
**Histopathological findings**	
Lymphoma	68
High-grade glioma (WH0 grade 3–4)	113
Low-grade glioma (WHO grade 1–2)	58
Other neuroepithelial tumors	33
Inflammatory diseases	45
Metastasis	11
Cerebral infarction	9
Papilloma of the choroid plexus	2
Germinoma	24
Demyelination	17
Necrotic tissue after radiotherapy	2
**Negative**	**25**
Normal brain tissue	3
Inconclusive	22

The bold values means the total of category item under each subheading.

### Demographic characteristics

The mean age of all 407 patients was 42.1 years (range, 6 months to 82 years). The mean age of the patients in the positive and negative diagnosis groups was 41.95 and 33.74 years, respectively. There were no significant differences in age between the two groups. There were also no significant sex-related differences in the diagnostic results, with 12 female and 13 male patients having a negative diagnosis (*p* = 0.27 and *p* = 0.33, respectively) (Table 2).

**Table 2:** Univariate analysis of factors affecting diagnosis result.

Variable indicators	Positive diagnosis	Negative diagnosis	p*	OR	95% CI	p**
Case number	382	25				
Age (mean ± S.D.)	41.95 ± 17	33.74 ± 17.9	0.21			
Gender						
Male (*n* = 233)	220	13	0.33			
Female (*n* = 174)	162	12	0.27	0.8	0.35–1.79	5.85 × 10^−1^
Biopsy location			0.98			
Midline/deep	202	6	0.12			
Suprasellar	6	4	0.03	0.04	0.01–0.20	4.99 × 10^−5^
Pineal	8	3	0.03	0.08	0.02–0.38	1.40 × 10^−3^
Superficial lobes	187	12	0.87	0.46	0.17–1.26	1.31 × 10^−1^
Whether the lesion is enhancing (yes/no)	281/101	20/5	0.01	0.70	0.25–1.90	4.79 × 10^−1^
Lesion volume ≤ 1 cm^3^	31	5	0.01	0.35	0.12–1.01	5.13 × 10^−2^
Biopsy method			0.79			0.79
ROSA (*n* = 35)	32	3	0.91			0.91
CAS-R (*n* = 65)	60	5	0.81	1.13	0.25–5.01	8.77 × 10^−1^
REMEBOT (*n* = 307)	290	17	0.67	1.6	0.44–5.76	4.72 × 10^−1^

*t-test; **chi-square test.

### Lesion location and size

The lesion distribution was as follows (multiple lesions or lesions involving both sides were counted according to the actual target site): supratentorial lobes, *n* = 170; deep supratentorial and midline areas (corpus callosum, basal ganglia, saddle area, and paraventricular area), *n* = 159; and infratentorial area, *n* = 78. In total, 198 lesions were sampled on the left and 209 on the right. In addition, 229 single lesions and 178 multiple lesions were sampled on the right side. There were 229 single lesions and 178 multiple lesions (Table 1). The volume of lesion was 13.42 cm^3^ (range, 0.31–88.9 cm^3^). In total, 301 lesions were significantly enhanced and 106 were not significantly enhanced. The lesion volume ranged from 1 to 67 cm^3^ (mean, 15 cm^3^). For the thirty-six lesions with a volume of ≤1 cm^3^, the diagnostic rate was 16.67%. In contrast, smaller lesion volumes were significantly associated with the negative diagnostic rate (*p* = 0.01), and the mean lesion volume in the positive diagnostic group (15.2 cm^3^) was larger than that in the negative diagnostic group (6.7 cm^3^). The average supratentorial (69.75 ± 2.31 mm) and sub-tentorial (92.07 ± 4.68 mm) trajectory length measured from the biopsy plan. With respect to location, there was no significant difference in the diagnostic rate between midline/deep and superficial lesions. Multifactorial analysis of the above factors showed a statistically significant difference in the effect of preoperative magnetic resonance enhancement of the lesion on the diagnostic rate (*t* = −2.32, *p* < 0.05), with an odds ratio of 0.70 (95% confidence interval, 0.25–1.90). A subgroup analysis of lesions located in the pineal and peri-saddle regions inferred a high negative diagnosis rate in 8 of 21 biopsies (*p* = 0.03) (Table 2).

### Biopsy procedure

There were no significant differences in demographics or lesion characteristics among the three groups. The mean procedure duration was 62.5 ± 17.3 min, and there were no significant differences in the diagnostic rates among the three modalities. The REMEBOT-based stereotactic biopsy had the shortest mean duration of 57.7 min and saved a significant amount of procedure time compared with the ROSA and CAS-R-2. As previously described, we did not find a significantly lower diagnostic rate for smaller lesions (≤1 cm^3^). However, a subgroup analysis showed that even in small-lesion biopsies, the CAS-R-2 and REMEBOT groups had higher diagnostic rates (85 and 92% for lesions of ≤1 cm^3^, respectively). In addition, positive diagnostic results were obtained in the five patients who underwent a second stereotactic biopsy.

## Discussion

Because of the complexity of occupying intracranial lesions, radiological examinations based on morphological imaging alone have a 20%–30% misdiagnosis rate for intracranial lesions compared with histological diagnosis (1), and such examinations introduce many uncertainties (2, 8). Stereotactic biopsy is used to obtain tissue from intracranial lesions for pathological diagnosis and establishment of treatment strategies (2, 8, 9). Various factors, including the patient’s age and sex and the lesion volume and location, may influence the diagnostic rate of stereotactic biopsy of brain lesions (4, 10). A diagnostic rate of >90% is usually acceptable (11). The diagnostic rate is influenced by the expertise, relevant case experience, and qualifications of the staff performing brain biopsies in each clinical center (12). The diagnostic rate of 93.86% in the present study is consistent with that obtained in one of the most significant international retrospective studies of 4,589 patients who underwent 5,000 biopsies (11). When stereotactic biopsies are performed by the same neurosurgeons with the same qualifications, as in the present study, the stability of the human surgical operation can be guaranteed. The rate of all postoperative complications was <1%, and no complications such as death, epilepsy, neurological deficits, or infection occurred. Three of the four complications were bleeding at the biopsy site and one was cerebral edema, and only one patient required a secondary surgical intervention. The average operative time was <65 min, and the safety and efficiency of the procedure were better than those in most reports in the domestic and international literature (2, 11, 12).

In the present study, the patients’ age and sex were not significantly associated with the diagnostic rate, which is consistent with the results of most other studies (4, 8, 13, 14). However, differences in the rate of definitive diagnosis by biopsy between older and younger patients have been reported (10, 15). For example, when an age of 40 years was set as the cut-off point in one study, the diagnostic rate was 75.9% in the younger group (<40 years of age) and 90.6% in the older group (>40 years of age) (10). However, this trend was not found in our patients.

Some studies have shown a positive association between lesions located deep in the brain and the negative diagnostic biopsy rate (16). Such findings suggest that the lesion location is a significant influencing factor of the diagnostic rate (10, 17–19). In other studies, however, the lesion location proved irrelevant (10, 13, 15, 20, 21). The depth of the lesions in our study did not affect the diagnostic rate. Similar to our report, another study showed that the target path depth had no effect on the success rate of stereotactic biopsy (9, 18). In our subgroup stratified analysis, we found that lesions in the suprasellar area and pineal regions might have a higher rate of negative diagnosis. This might occur if the biopsy needle punctures the brain pool or ventricles during tissue sampling, resulting in cerebrospinal fluid aspiration. This harmful loss of aspiration pressure can lead to tissue loss into the Sedan needle hole. As a result, sampling errors can occur.

According to several studies, the lesion size is another vital influencing factor for the diagnostic result of brain biopsies (14, 19). Smaller lesions are more likely to produce negative results, and larger lesions are more likely to produce positive results. Our study findings are consistent with this. However, other studies have shown that lesion size is not relevant to the diagnostic result (10, 20). Our experience using the REMEBOT and CAS-R-2 compared with the ROSA showed differences in the mean lesion volume of ≤l cm^3^. In general, using bone nail-based marking and auto-registration methods for deep and small lesions is recommended to reduce systematic errors (17, 20, 22).

For a considerable period of clinical practice, frame-based brain biopsy was considered the gold standard for stereotactic intracranial lesion biopsy. However, a recent study proved no difference in diagnostic rates or complications between frame-based and frameless stereotactic surgery (11). Our study demonstrated no significant difference in the diagnostic rates among the three fameless procedures. The obvious advantage of the REMEBOT-guided procedure is that it takes less registration time than the ROSE and CAS-R-2 procedures. Similar results have been reported from other centers (10, 14).

Another factor affecting the diagnostic rate of biopsies is the surgeon’s experience. The literature states that the operator’s technical skill level could be a key factor affecting the diagnostic rate of biopsy. Differences in the quantity of similar surgeries performed, proficiency, and expertise among centers are all essential factors affecting accuracy (23). More experienced surgeons are a significant predictor of favorable diagnostic rates; i.e., more senior neurosurgeons have higher diagnostic rates than less senior neurosurgeons. Our study minimized human influence. However, we hypothesize that personal experience is a confounding factor for accuracy and positive diagnostic rates. For this reason, we have organized an expert consensus on biopsy to reduce human factors and bias in biopsy procedure protocols (10, 24–26).

This study has three main limitations. First, it was a nonrandomized single-center study. Second, several pathologists were involved in evaluating the biopsy specimens, which may have affected the diagnostic rate. Third, the number of cases was relatively low and we designed the puncture path to avoid passing through the arachnoid cisterna of the sulci but did not take into account the trajectory crosses a ventricular surface, which may be as another factor influencing the diagnostic accuracy (27, 28).

In conclusion, to the best of our knowledge, this is the most extensive study of different types of robot-guided stereotactic intracranial lesion biopsies in China. This summary of our 3 years of experience related to brain biopsies at a single center involving different neurosurgical robot-assisted stereotactic operating systems is clinically valuable because it reflects routine real-world practice and identified the factors that affect diagnostic rates. The different procedures had high safety and effectiveness (3). We consider the lesion location and size, the number of biopsies performed at the center, and the relevant experience of the surgeon to be essential factors influencing the diagnostic rate and the safety and efficacy of robot-guided stereotactic intracranial lesion biopsies. In contrast, patient age, patient sex, surgical method, and lesion depth are not statistically associated with positive diagnostic rates.

## Data availability statement

The original contributions presented in the study are included in the article/supplementary material, further inquiries can be directed to the corresponding authors.

## Ethics statement

The studies involving human participants were reviewed and approved by the Xuanwu Hospital Ethics Committee. Written informed consent to participate in this study was provided by the participants’ legal guardian/next of kin. Written informed consent was obtained from the individual(s), and minor(s)’ legal guardian/next of kin, for the publication of any potentially identifiable images or data included in this article.

## Author contributions

YF, WY, SY, WP, WH, FX, WC, CS, and ZG listed have participated in the experimental design. YF and WY collected the data. YF finished the paper. All authors contributed to the article and approved the submitted version.

## Conflict of interest

The authors declare that the research was conducted in the absence of any commercial or financial relationships that could be construed as a potential conflict of interest.

## Publisher’s note

All claims expressed in this article are solely those of the authors and do not necessarily represent those of their affiliated organizations, or those of the publisher, the editors and the reviewers. Any product that may be evaluated in this article, or claim that may be made by its manufacturer, is not guaranteed or endorsed by the publisher.

## References

[1] Arbit E Galicich JH. Importance of image-guided stereotactic biopsy to confirm the diagnosis in an oncological setting. Ann Surg Oncol. (1994) 1:368–72. doi: 10.1007/BF02303807 7850537

[2] Zengmin Tian Yaming Wang. Stereotactic brain biopsy technique. Beijing: People's Military Medical Press, (2012):32–35.

[3] Bex A Mathon B. Advances, technological innovations, and future prospects in stereotactic brain biopsies. Neurosurg Rev. (2023) 46:5. doi: 10.1007/s10143-022-01918-w PMC9734929 36471144

[4] Dammers R Haitsma IK Schouten JW Kros JM Avezaat CJ Vincent AJ. Safety and efficacy of frameless and framebased intracranial biopsy techniques. Acta Neurochir. (2008) 150:23–9. doi: 10.1007/s00701-007-1473-x, PMID: 18172567

[5] Hu Y Cai P Zhang H Adilijiang A Peng J Li Y. A Comparation between frame-based and robot-assisted in stereotactic biopsy. Front Neurol. (2022) 13:928070. doi: 10.3389/fneur.2022.928070, PMID: 35923834 PMC9339900

[6] Cheng G Yu X Zhao H Cao W Li H Li Q. Complications of stereotactic biopsy of lesions in the sellar region, pineal gland and brainstem: a retrospective, single-center study. Medicine. (2020) 99:e18572. doi: 10.1097/MD.0000000000018572 32080071 PMC7034708

[7] Li C Wu S Huang K Li R Jiang W Wang J. Comparison of the safety, efficacy, and accuracy of frame-based versus Remebot robot-assisted stereotactic Systems for Biopsy of brainstem tumors. Brain Sci. (2023) 13:362. doi: 10.3390/brainsci13020362, PMID: 36831906 PMC9954386

[8] Qiao Liang Y Tao LY. Application status of surgical robot in neurosurgery. Chin J Neurosurg. (2020) 36:1286–9. doi: 10.3760/cma.j.cn112050-20200825-00468

[9] Johannes Tilgner MD Manfred Herr MD Christoph Ostertag MD Benedikt Volk MD. Validation of intraoperative diagnoses using smear preparations from stereotactic brain biopsies: intraoperative versus final diagnosis—influence of clinical factors. Neurosurgery. (2005) 56:257–65. doi: 10.1227/01.neu.0000148899.39020.87, PMID: 15670374

[10] Shiqiang W Junwen W Pan G Liu W Hu F Jiang W. A comparison of the efficacy, safety, and duration of frame-based and remebot robot-assisted frameless stereotactic biopsy. Br J Neurosurg. (2020) 35:319–23. doi: 10.1080/02688697.2020.1812519 32940070

[11] Dhawan S He Y Bartek J Jr Alattar AA Chen CC. Comparison of frame-based versus frameless intracranial stereotactic biopsy: systemic review and meta-analysis. World Neurosurg. (2019) 127:607–616.e4. doi: 10.1016/j.wneu.2019.04.016, PMID: 30974279

[12] Bik LL Kugan V Donald NSL Albert SHW. Factors afecting diagnostic yield in stereotactic biopsy for brain lesions: a 5-year single-center series. Neurosurg Rev. (2021) 45:1473–80. doi: 10.1007/s10143-021-01671-6 34628562

[13] Kim JE Kim DG Paek SH Jung HW. Stereotactic biopsy for intracranial lesions: reliability and its impact on the planning of treatment. Acta Neurochir. (2003) 145:547–55. doi: 10.1007/s00701-003-0048-8, PMID: 12910397

[14] Lu Y Yeung C Radmanesh A Wiemann R Black PM Golby AJ. Comparative efectiveness of frame-based, frameless, and intraoperative magnetic resonance imaging-guided brain biopsy techniques. World Neurosurg. (2015) 83:261–8. doi: 10.1016/j.wneu.2014.07.043, PMID: 25088233 PMC4450019

[15] Ferreira MP Ferreira NP Pereira Filho Ade A Pereira Filho Gde A Franciscatto AC. Stereotactic computed tomography-guided brain biopsy: diagnostic yield based on a series of 170 patients. Surg Neurol. (2006) 65 27–1:32:S27–32. doi: 10.1016/j.surneu.2005.11.036, PMID: 16427444

[16] Livermore LJ Ma RC Bojanic S Pereira Erlick AC. Yield and complications of frame-based and frameless stereotactic brain biopsy – the value of intra-operative histological analysis. Br J Neurosurg. (2014) 28:637–44. doi: 10.3109/02688697.2014.887657, PMID: 24568533

[17] Riche M Amelot A Peyre M Capelle L Carpentier A Mathon B. Complications after frame-based stereotactic brain biopsy: a systematic review. Neurosurg Rev. (2021) 44:301–7. doi: 10.1007/s10143-019-01234-w 31900737

[18] Tsermoulas G Mukerji N Borah AJ Mitchell P Ross N. Factors affecting diagnostic yield in needle biopsy for brain lesions. Br J Neurosurg. (2013) 27:207–11. doi: 10.3109/02688697.2012.722239, PMID: 22984980

[19] Woodworth GF McGirt MJ Samdani A Garonzik I Olivi A Weingart JD. Frameless image-guided stereotactic brain biopsy procedure: diagnostic yield, surgical morbidity, and comparison with the frame-based technique. J Neurosurg. (2006) 104:233–7. doi: 10.3171/jns.2006.104.2.233, PMID: 16509497

[20] Owen CM Linskey ME. Frame-based stereotaxy in a frameless era: current capabilities, relative role, and the positive-and negative predictive values of blood through the needle. J Neuro-Oncol. (2009) 93:139–49. doi: 10.1007/s11060-009-9871-y 19430891

[21] Zanello M Roux A Debacker C Peeters S Edjlali-Goujon M Dhermain F. Postoperative intracerebral hematomas following stereotactic biopsies: poor planning or poor execution. Int J Med Robot. (2021) 17:e2211. doi: 10.1002/rcs.2211 33345461

[22] Maragkos GA Penumaka A Ahrendsen JT Salem MM Nelton EB Alterman RL. Factors affecting the diagnostic yield of frame-based stereotactic intracranial biopsies. World Neurosurg. (2020) 135:e695–701. doi: 10.1016/j.wneu.2019.12.102, PMID: 31883483

[23] Li Z Zhang JG Ye Y Li X. Review on factors afecting targeting accuracy of deep brain stimulation electrode implantation between 2001 and 2015. Stereotact Funct Neurosurg. (2016) 94:351–62. doi: 10.1159/000449206 27784015

[24] Functional Neurosurgery Group of Neurosurgery Society of Chinese Medical Association. Functional neurosurgery Group of Neurosurgery Society of Chinese medical doctor association, expert steering Committee of National Robot Application Demonstration Project for neurosurgery. Chinese expert consensus on stereotaxic intracranial biopsy, 2021 edition. Chin J Med. (2021) 101:3534–41.

[25] Liu H-G Liu Y-Y Zhang H Meng F-G Zhang K Zhu G-Y. A bulk retrospective study of robot-assisted stereotactic biopsies of intracranial lesions guided by Videometric tracker. Front Neurol. (2021) 12:682733. doi: 10.3389/fneur.2021.682733, PMID: 34421791 PMC8371178

[26] Ma F-Z Liu D-F Yang A-C Zhang K Meng F-G Zhang J-G. Application of the robot-assisted implantation in deep brain stimulation. Front Neurorobot. (2022) 16:996685. doi: 10.3389/fnbot.2022.996685, PMID: 36531913 PMC9755501

[27] Zanello M Roux A Debacker C Peeters S Edjlali-Goujon M Dhermain F. Postoperative intracerebral haematomas following stereotactic biopsies: poor planning or poor execution? Int J Med Robot. (2021) 17:e2211. doi: 10.1002/rcs.2211 33345461

[28] Katzendobler S Do A Weller J Dorostkar MM Albert NL Forbrig R. Diagnostic yield and complication rate of stereotactic biopsies in precision medicine of gliomas. Front Neurol. (2022) 13:822362. doi: 10.3389/fneur.2022.822362, PMID: 35432168 PMC9005817

